# Human stem cell-derived retinal epithelial cells activate complement via collectin 11 in response to stress

**DOI:** 10.1038/s41598-017-15212-z

**Published:** 2017-11-07

**Authors:** Giorgia Fanelli, Anai Gonzalez-Cordero, Peter J. Gardner, Qi Peng, Milan Fernando, Magdalena Kloc, Conrad A. Farrar, Arifa Naeem, Peter Garred, Robin R. Ali, Steven H. Sacks

**Affiliations:** 1 MRC Centre for Transplantation, King’s College London and Guy’s and St Thomas’ NHS Trust, Guy’s Hospital, London, UK; 2 UCL Institute of Ophthalmology, London, UK; 3 Laboratory of Molecular Medicine, Department of Clinical Immunology Section 7631 Rigshospitalet, University of Copenhagen, Copenhagen, Denmark

## Abstract

Age-related macular degeneration (AMD) is a major cause of blindness and is associated with complement dysregulation. The disease is a potential target for stem cell therapy but success is likely to be limited by the inflammatory response. We investigated the innate immune properties of human induced-pluripotent stem cell (iPSC)-derived RPE cells, particularly with regard to the complement pathway. We focused on collectin-11 (CL-11), a pattern recognition molecule that can trigger complement activation in renal epithelial tissue. We found evidence of constitutive and hypoxia-induced expression of CL-11 in iPS-RPE cells, and in the extracellular fluid. Complement activation on the cell surface occurred in conjunction with CL-11 binding. CL-11 has been shown to activate inflammatory responses through recognition of L-fucose, which we confirmed by showing that fucosidase-treated cells, largely, failed to activate complement. The presence of CL-11 in healthy murine and human retinal tissues confirmed the biological relevance of CL-11. Our data describe a new trigger mechanism of complement activation that could be important in disease pathogenesis and therapeutic interventions.

## Introduction

The retinal pigment epithelium (RPE) consists of a monolayer of cells situated between the photoreceptor cells and the choroid and plays a critical role in the visual cycle, maintaining the health of photoreceptor cells by providing nutrients, growth factors and by continually phagocytosing photoreceptor outer segment discs. Together with Bruch’s membrane, tight junctions between neighboring RPE cells form the outer blood-retinal barrier, which is essential for maintaining retinal homeostasis. Loss of RPE cells and the subsequent loss of photoreceptor cells they support is associated with degenerative diseases such as Stargardt’s disease, retinitis pigmentosa and age-related macular degeneration (AMD), the leading cause of blindness in the developed world^1^. Current therapies for AMD are only effective in reducing aberrant blood vessel formation in neovascular AMD and there is no therapy for geographic atrophy, an advanced non-vascular form that comprises a third of all late-stage AMD patients^2^. A growing body of evidence suggests that choroidal blood flow is reduced in AMD^3,4^ and data from transgenic mouse models where HIF (hypoxia-inducible factor) pathways are specifically activated in RPE show photoreceptor degeneration and features consistent with some aspects of AMD pathology^5^. As HIF pathways are linked to inflammation^6^ it is possible that some of the chronic dysregulation of local para-inflammatory responses in the eye associated with AMD^7–9^ may be driven by RPE hypoxic stress resulting in the aberrant activation of complement on host cells. Regrettably, how complement system dysregulation in retina can lead to cell and tissue damage under inflammatory conditions, including AMD, has not yet been addressed. Circulating levels of C3a, C3d and C5a, have been found in AMD patients^10,11^ indicating enhanced local complement activation. Moreover, polymorphisms in a number of complement genes such as, CFH, C3, CFB and C2, have been shown to be associated with AMD^12–14^ suggesting that the complement system, in particular the alternative pathway, may be dysregulated in AMD patients. Therefore, appropriate control of local complement activation may preserve retinal structure and function. Since RPE loss is a major component of AMD pathogenesis, there is major interest in the development of treatment strategies involving the replacement of this monolayer by grafting healthy RPE under the macula^1^. A number of studies have demonstrated preservation of visual function following the transplantation of stem cell–derived RPE into animal models of retinal degeneration^15,16^. The trials to date suggest that the transplanted cells are well tolerated and are not tumorogenic^17–19^. Although the eye is immune privileged, this is likely to provide a limited advantage for RPE transplantation and thus further studies are required to determine whether and under what circumstances the cells might provoke host immune responses.

Collectin 11 (CL-11, also known as collectin-kidney 1 or CL-K1 and is encoded by *COLEC11*) is a recently described soluble C-type lectin of innate defence^20,21^. It belongs to the group of collectins that include the surfactant proteins SP-A and SP-D, mannose-binding lectin (MBL), collectin liver 1 (CL-10 or CL-L1) and collectin placenta 1 (CL-12 or CL-P1)^22,23^. The structure and function of CL-11 are similar to MBL regarding the binding to carbohydrates on pathogens and activating MBL-associated serine proteases (MASPs) to initiate the lectin pathway of complement activation^21,24^, resulting in the generation of complement activation products including the membrane attack complex (MAC) formation and targeted cell death. An important difference, however, is that while the large macromolecular complexes of MBL in the serum are mainly produced by hepatic synthesis^25^, CL-11 is primarily generated by parenchymal cells in the extravascular compartment^26,27^. It has been recently demonstrated that CL-11 activates a destructive inflammatory response through recognition of L-fucose on post-ischemic renal tubule cells close to the site of CL-11 production^28^. Although the adrenal glands, kidney and liver are the main expression sites of human CL-11^27,29^, moderate levels of CL-11 were also found in the ovaries, testis and retina^20^. In this study we investigated the role of CL-11 on human iPS-RPE cells by examining the interplay between cell hypoxia and local induction of CL-11 binding and complement activation on the hypoxia-stressed cells. A more detailed knowledge of this relationship may provide insights for understanding the potential inflammatory and immune responses of the host environment to stem cell derived-RPE cells following transplantation.

## Results

### CL-11 is expressed in the human eye with similar expression found in iPS-RPE cells as in endogenous RPE

**Figure 1:**
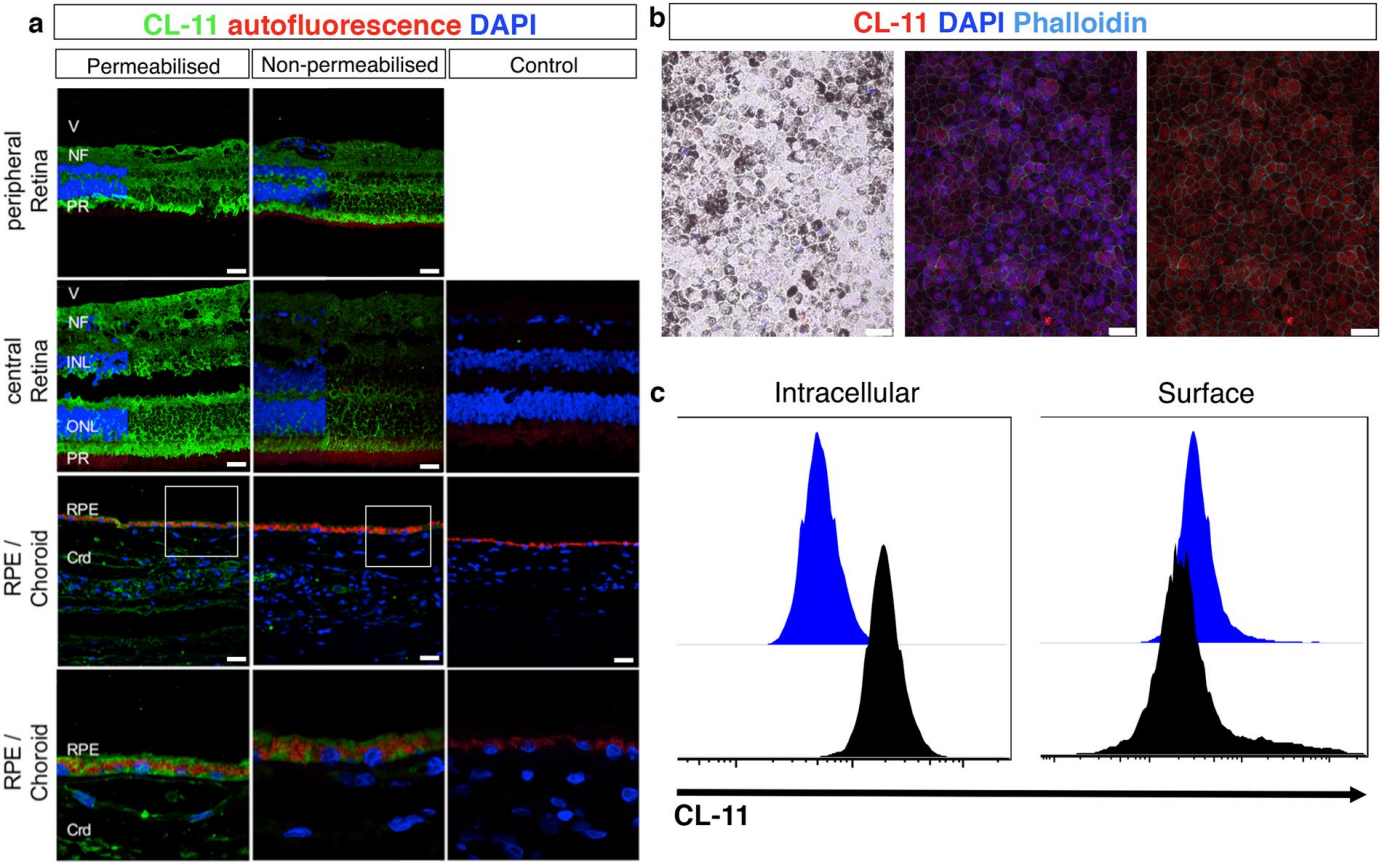
Human retina tissue expression and iPS-RPE cell expression patterns of CL-11. (**a**) Representative confocal image showing CL-11 expression on permeabilised (left panel) and non-permeabilised (middle panel) human retinal sections. The sections shown are taken from the peripheral retina and from the central retina outside the macula. Control sections were incubated with no primary antibody (right panel). CL-11 (green), nuclei (blue) and autofluorescence (red) are shown. Scale bars, 25 μm. CL-11 staining without DAPI is shown on the right side of peripheral retina and central retina panels. High magnification images of area indicated in the white box are shown in the bottom panel. Scale bar, 10 μm. (**b**) Representative confocal microscopic image showing differentiated iPS-RPE cells morphology (left panel) and CL-11 staining on permeabilised human iPS-RPE cells (middle and right panel). CL-11 (red), nuclei (blue) and phalloidin (aqua) are shown. Scale bars, 25 μm (**c**) Flow cytometry histograms showing CL-11 expression on permeabilised iPS-RPE cells (intracellular) versus non-permeabilised cells (surface). Cells stained with secondary antibody alone were used as control.

Although transcription of CL-11 has been reported in the human retina^20^, its expression in the eye has not been explored in detail. Here, we investigated the expression pattern of CL-11 on human retinal sections by confocal microscopy. The specificity of the anti-human CL-11 antibody was validated by immunohistochemical staining of *Colec11* ^+/+^ and *Colec11* ^−/−^ mouse eye sections (Supplemental Fig. 1a). As illustrated in Fig. 1a, permeabilisation to allow detection of both surface-bound and intracellular CL-11 revealed staining throughout several of the retinal layers, in peripheral as well as central retinal regions. Expression of CL-11 was particularly strong in the photoreceptor inner segments, in the cell inner and outer plexiform layers and in the nerve fibre layer of the ganglion cell layer. Positive labelling for CL-11 above background autofluorescence of pigment was also evident in the RPE layer and in the choroid, best seen at the higher magnification. Cell surface staining of CL-11 was present but fainter throughout the retina and RPE suggesting a predominant intracellular expression pattern (Fig. 1a), whereas within the choroid CL-11 was largely absent from the surface of cells. In contrast to the retina, intense CL-11 staining was observed in the anterior uvea section, iris and ciliary body, on both permeabilised and non-permeabilised sections (Supplemental Fig. 2).

Having detected CL-11 expression in the eye, we then determined whether CL-11 was also present on differentiated human iPS-RPE cells, a potential source for RPE replacement in patients with AMD and inherited retinal degeneration. As shown in Fig. 1b, cultured cells displayed the classic cobblestone pigmented morphology. Following permeabilisation, the iPS-RPE cells grown under steady state conditions displayed a pronounced intracellular staining pattern for CL-11 (Fig. 1b,c), whereas the expression on the cell surface was markedly lower (Fig. 1c). The same pattern of expression was observed on primary cultures of adult murine RPE cells (Supplemental Fig. 1b,c). Our data confirm that CL-11 is expressed in the human and mouse eye and reveal that human iPS-RPE cells show a pattern of surface and intracellular expression of CL-11 that is similar to adult RPE.

### Effect of hypoxic stress on cultured human iPS-RPE cells

**Figure 2:**
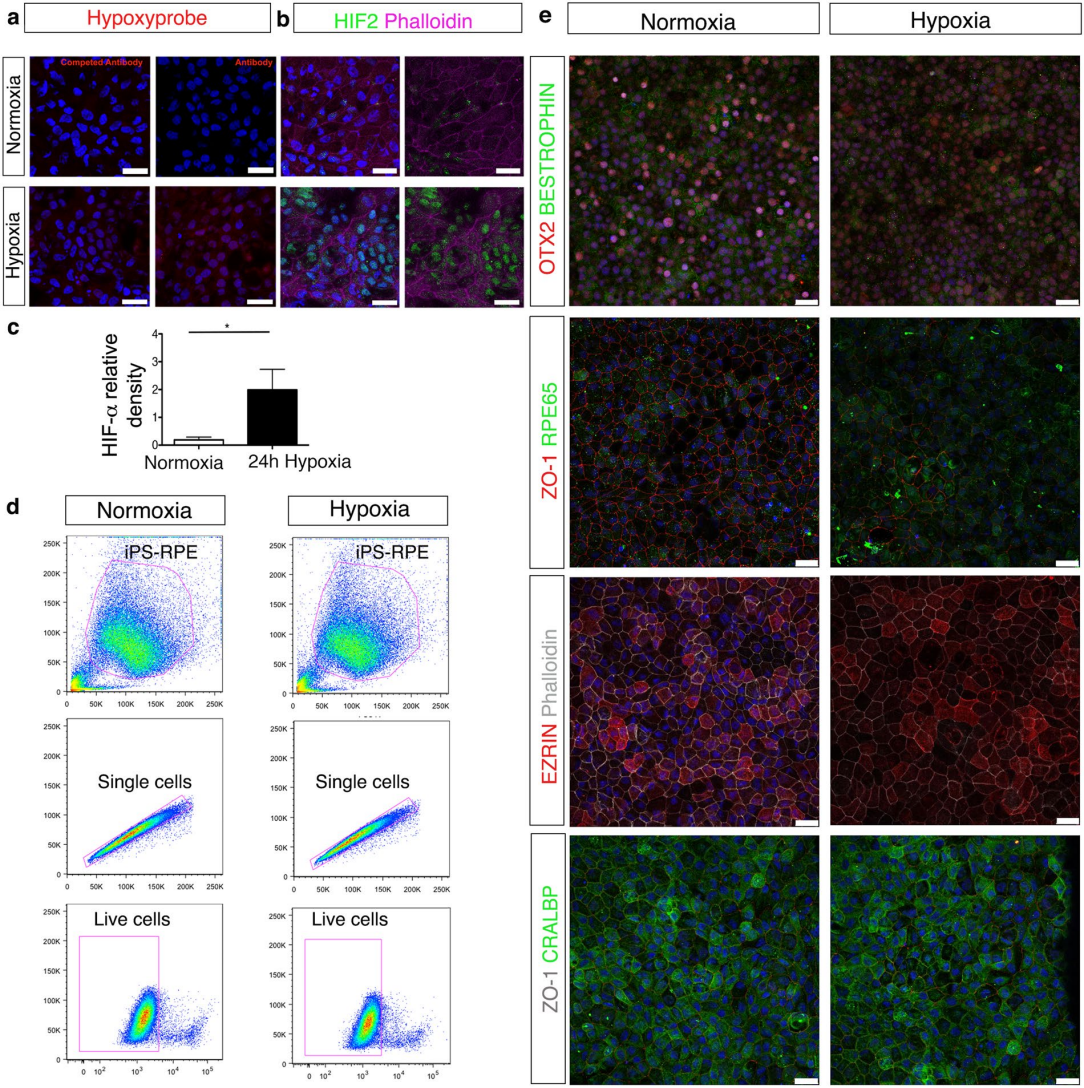
iPS-RPE cell viability and phenotype following hypoxic stress. (**a**) Immunohistochemistry images showing RPE cells treated with hypoxyprobe and antibody stained for probe detection. A competed antibody (background staining) was used as control. (**b**) Images showing iPS-RPE cells stained for HIF2 in normoxia and hypoxia conditions. Nuclei are counterstained with DAPI (blue). Scale bars, 25 μm (**c**) Western blot analysis showing up-regulation of HIF-1α following 24 hours  of hypoxic stress compared to non-stressed cells. N = 5 independent experiments *P < 0,05, *t*-test. Full-length blots are presented in Supplemental Fig. 3. (**d**) Representative FACS plots showing the viability of iPS-RPE cells following or not hypoxic stress using the Live/Dead assay. (**e**) Representative confocal images showing iPS-RPE phenotype following or not hypoxic stress. Cells were stained for: OTX2, Bestrophin, ZO-1, RPE65, EZRIN, CRALBP and Phalloidin. Scale bars, 25 μm.

The concentration of oxygen in human tissues varies from 3 to 5% and a decline below this range is considered as hypoxia^30^. In macular degeneration, progression of disease is associated with hypoxia due to disruption in the underlying choroid and perturbations in retinal blood flow^31,32^. Therefore we sought to develop an *in vitro* model of hypoxia-induced stress on cultured human iPS-RPE cells. To induce hypoxia, we maintained cultured iPS-RPE cells in 1% oxygen in a controlled chamber for 24 hours. We first confirmed that the cells were hypoxic. Immunofluorescence analysis showed positive nuclear staining with a hypoxia-specific probe (Fig. 2a) and up-regulation of the hypoxia-inducible factor HIF2 under hypoxic conditions (Fig. 2b). Furthermore, western blot analysis demonstrated a significant increase in HIF-1α confirming that iPS-RPE cells were sensitive to hypoxic stress (Fig. 2c). Prolonged hypoxia can lead to cell death, thus we assessed the viability of the cells cultured under normal and hypoxic conditions following 24-hours hypoxia^33^. Flow cytometry analysis showed no discernable difference in cell death in both of these conditions (Fig. 2d). Finally, we checked the morphological appearance and the presence of RPE-specific markers following hypoxic stress. No major changes were observed in the hypoxic iPS-RPE cells. The typical cobblestone RPE morphology was still intact as demonstrated by staining of the ZO-1 tight junction protein. The expression of BESTROPHIN and OTX2 decreased following hypoxic stress whereas no noticeable differences were observed in the other RPE markers tested (Fig. 2e).

### Hypoxia-stressed iPS-RPE cells show increased expression, secretion and L-fucose-mediated surface binding of CL-11

**Figure 3:**
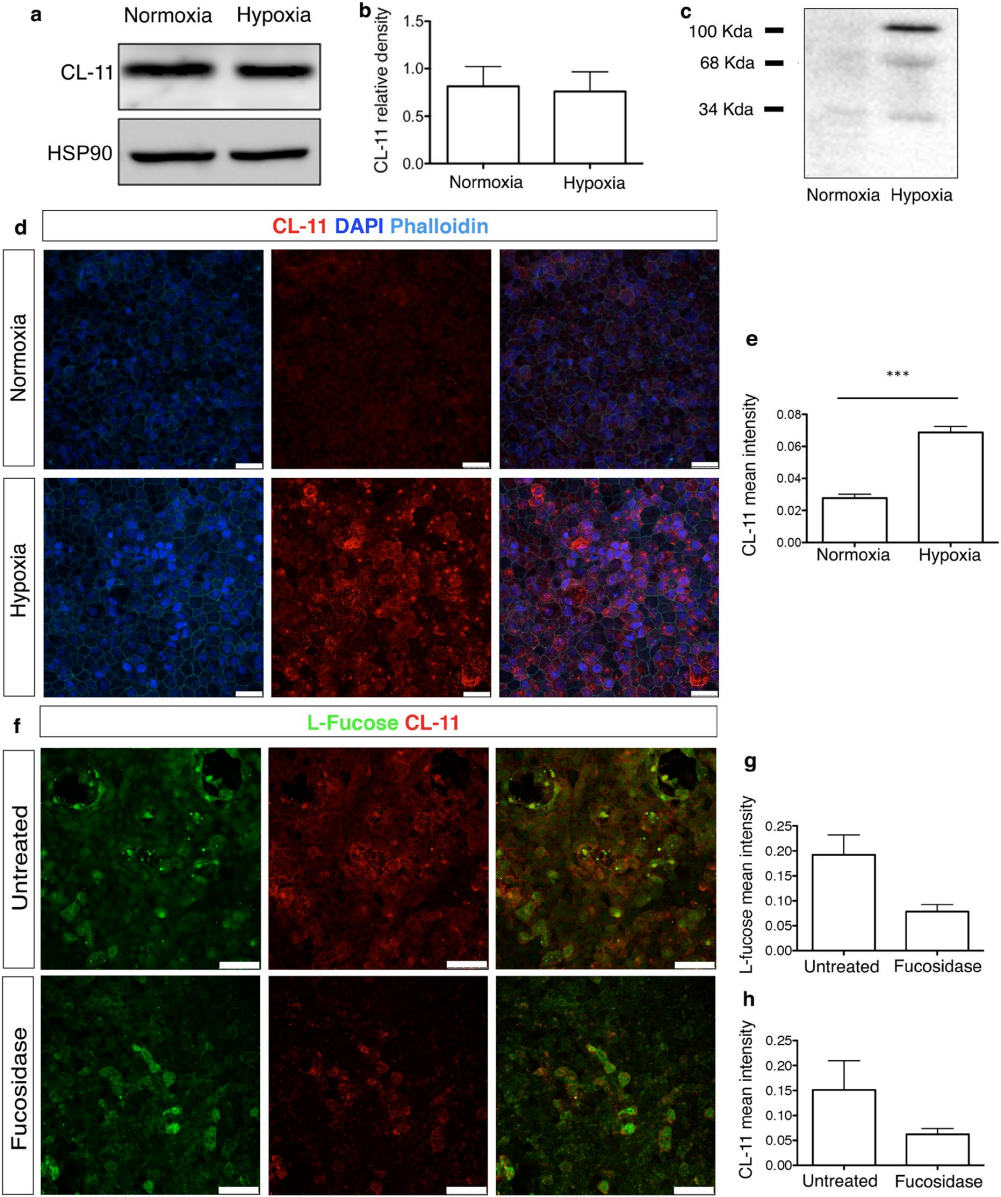
CL-11 binding to hypoxia-stressed iPS-RPE cells. (**a**) Representative Western blot under reducing conditions showing CL-11 expression in iPS-RPE cells under normal and hypoxic conditions. HSP90 was used as a loading control. Full-length blots are presented in Supplemental Fig. 4. (**b**) Relative density analysis of CL-11 in iPS-RPE cells following different treatments (N = 3 independent experiments). (**c**) Detection of both non-reduced (100 kDa) and reduced states (34 kDa) of CL-11 in the culture supernatants of iPS-RPE cells following or not hypoxic stress by Western blotting. (**d**) Representative immunofluorescence images showing CL-11 binding to the surface of hypoxic-stressed iPS- RPE cells compared to non-stressed cells. Scale bars, 25 μm. (**e**) Quantification of CL-11 binding in normal and hypoxic iPS-RPE cells using ImageJ software. CL-11 (red), nuclei (blue) and phalloidin (aqua) are shown. N = 3 independent experiments, ***P < 0,0001, *t*-test. (**f**) Confocal images of CL-11 (red) and L-fucose (green) showing co-expression of CL-11 and L-fucose (orange) on hypoxic–iPS-RPE cells treated or untreated with fucosidase. Scale bars, 50 μm. (**g**) Quantification of L-fucose expression and (**h**) CL-11 binding to hypoxia-stressed cells treated or untreated with fucosidase using ImageJ software. N = 4 independent experiments.

Adequate complement regulation in the eye is a *conditio sine qua non* to prevent tissue injury^34^. However, alteration in the expression of complement regulators on the surface of RPE cell line can occur following cellular stress^35^. In the present study, we examined whether hypoxic stress could affect the expression of CL-11 in human iPS-RPE cells and whether this led to increased CL-11 binding on the surface of these cells. Western blot analysis showed that the amount of CL-11 protein in iPS-RPE cells following hypoxia was comparable to those cells cultured under normal level of oxygen (Fig. 3a,b). However, soluble CL-11 was detectable in the supernatants of hypoxic iPS-RPE cells (Fig. 3c) suggesting CL-11 release following hypoxic cell stress. To investigate the presence of CL-11 at the surface of iPS-RPE cells, we performed immunofluorescence staining of CL-11 on non-permeabilised iPS-RPE cells under normal or hypoxic conditions, as previously described. We observed that CL-11 detection was significantly higher on the surface of hypoxia–stressed iPS-RPE cells compared to normoxic cells (Fig. 3d,e) suggesting that iPS-RPE cells are able to produce, secrete and bind CL-11 following hypoxic stress.

The binding of CL-11 to renal tubular epithelial cell surface is mediated through the recognition of L-fucose, the preferred ligand for both human and murine CL-11^20,26,29^. As it has been shown that retinal pigment epithelium contains glycoconjugates rich in α-Fuc^36^, we examined whether L-fucose could be recognised by CL-11 on stressed-iPS-RPE cells. The merged immunofluorescence image in Fig. 3f shows co-localization of CL-11 and L-fucose on the surface of non-permeabilised hypoxia-stressed iPS-RPE cells, suggesting that L-fucose is bound in an autocrine manner by CL-11 secreted by stressed iPS-RPE cells. To verify that L-fucose acts as a ligand for CL-11, we treated human iPS-RPE cells with fucosidase to specifically remove L-fucose from the cell surface. As expected, the fucosidase-treated cells showed a dramatic decrease of L-fucose expression and CL-11 binding confirming the co-localization of these two molecules on hypoxia-stressed iPS-RPE cells (Fig. 3f,g,h).

### Complement activation triggered by CL-11 results in C3 binding and MAC detection on hypoxia-stressed iPSC-RPE cells

**Figure 4:**
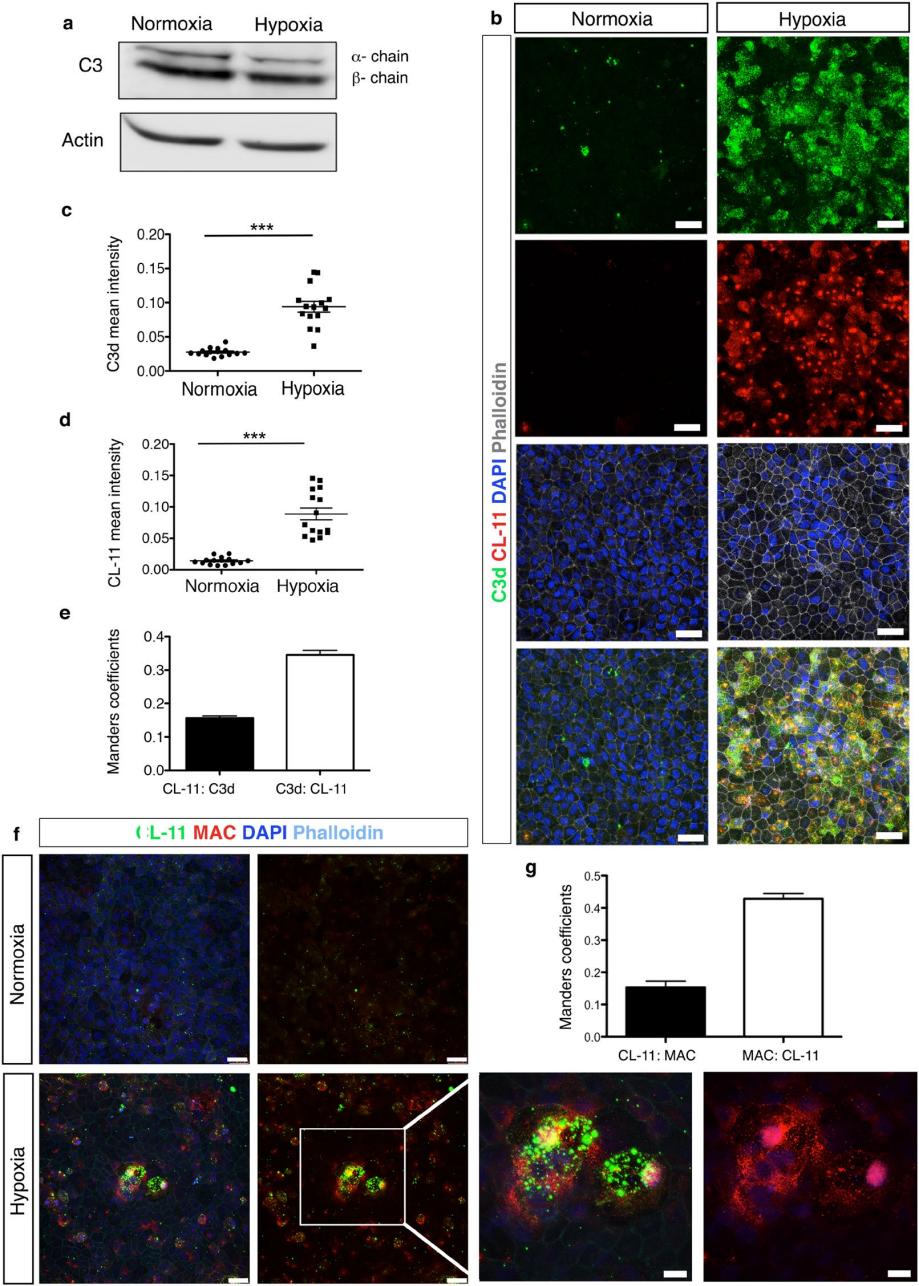
C3d deposition and Mac assembly on hypoxia-stressed iPS-RPE cells. (**a**) Representative Western blot showing C3 protein expression in iPS-RPE cells under normal and hypoxic conditions. Actin was used as a loading control. Full-length blots are presented in Supplemental Fig. 5. (**b**) Representative immunofluorescence images showing CL-11 (red) and C3d (green) staining on hypoxia-stressed iPS- RPE cells compared to non-stressed cells. Nuclei (blue) and phalloidin (grey) are shown. Scale bars, 25 μm. Quantification of CL-11 (**c**) and C3d (**d**) expression following or not hypoxia stress using ImageJ software. N = 3 independent experiments; n = 15 images analysed ***P < 0,0001, *t*-test. (**e**) CL-11 and C3d co-localization was quantified by calculating Mander’s co-localization coefficients using JACoP plug-in and ImageJ software. (**f**) Representative confocal images of CL-11 (green), MAC (red) nuclei (blue) and phalloidin (aqua) showing co-expression of CL-11 and MAC on hypoxic iPS-RPE compared to non-stressed cells. Scale bars, 25 μm. High magnification image (white box) showing co-localization of CL-11 and MAC. Scale bars, 10 μm. (**g**) CL-11 and MAC co-localization was quantified by calculating Mander’s co-localization coefficients using JACoP plug-in and ImageJ software. N = 3 independent experiments.

Epithelial cells from different organs can respond to cellular stress in an idiosyncratic manner. In fact, aberrant complement activation on epithelial barrier layers is a hallmark of inflammatory diseases in several organs, including the kidney^37^, the lung^38^ and the eye^39^. Therefore, we investigated whether locally produced CL-11 was able to initiate complement activation on iPS-RPE cells following hypoxia-induced cell stress. We measured the expression of the central complement component C3 in iPS-RPE cells grown in the absence of foetal bovine serum under normal or hypoxic conditions. Although we did not detect any major changes in C3 protein (both α-chain and β-chain) expression in non-hypoxic versus hypoxic iPS-RPE cells (Fig. 4a), immunofluorescence analysis revealed a significant increase of the activated fragment C3d, formed after cleavage of C3, on the surface of hypoxia-stressed iPS-RPE cells (Fig. 4b,c). Under hypoxic conditions, high levels of CL-11 were observed on the cell surface as well C3d deposition (Fig. 4d). C3d was mainly co-localised with CL-11 on the surface (Fig. 4b). Quantification of the co-localization of C3d with CL-11 using Mander’s co-localization coefficient showed clearly the level of association of two molecules on the cell surface (Fig. 4e). This result highlights the relationship of these two molecules indicating strong complement deposition at the site of CL-11 binding. Of interest, many of the bright spots corresponding to strong areas of C3d and CL-11 co-localisation are surrounded by a more diffuse punctate pattern for C3d staining, which could suggest complement activation is triggered at focal sites where CL-11 is bound and is amplified circumferentially by the alternative pathway in a CL-11 independent fashion.

In order to further characterize the pathophysiological role of CL-11, we tested whether this molecule under stress conditions was able to trigger membrane attack complex (MAC) formation on iPS-RPE cells. MAC, or C5b-9, is the terminal complex of the complement cascade that forms a lytic pore on the surface of target cell membranes inducing cell lysis and inflammatory responses^40–42^. In AMD, it is known that MAC assembles on RPE cells^43,44^ and the amount of MAC deposition is correlated with the loss of RPE cells^45,46^. Therefore, we tested the ability of iPS-RPE cells to produce the final component of the MAC complex, C9, under normal or hypoxic conditions by Luminex methodology. We detected low levels of C9 monomers only in the supernatants of the iPS-RPE cells cultured under normal conditions (data not shown). However, MAC positive staining was observed on hypoxia-stressed iPS-RPE cells compared to non-stressed cells suggesting that C9 molecules were incorporated into the terminal complex (Fig. 4f,g). Some areas showed co-localization of CL-11 with MAC whereas in other areas the relationship between MAC and CL-11 was less precise. Overall these results suggest that MAC formation on the iPS-RPE cells was triggered by hypoxia-induced CL-11 binding and complement activation.

### Removal of L-fucose ligand recognised by CL-11 results in less C3d deposition and MAC formation on stressed cells

**Figure 5:**
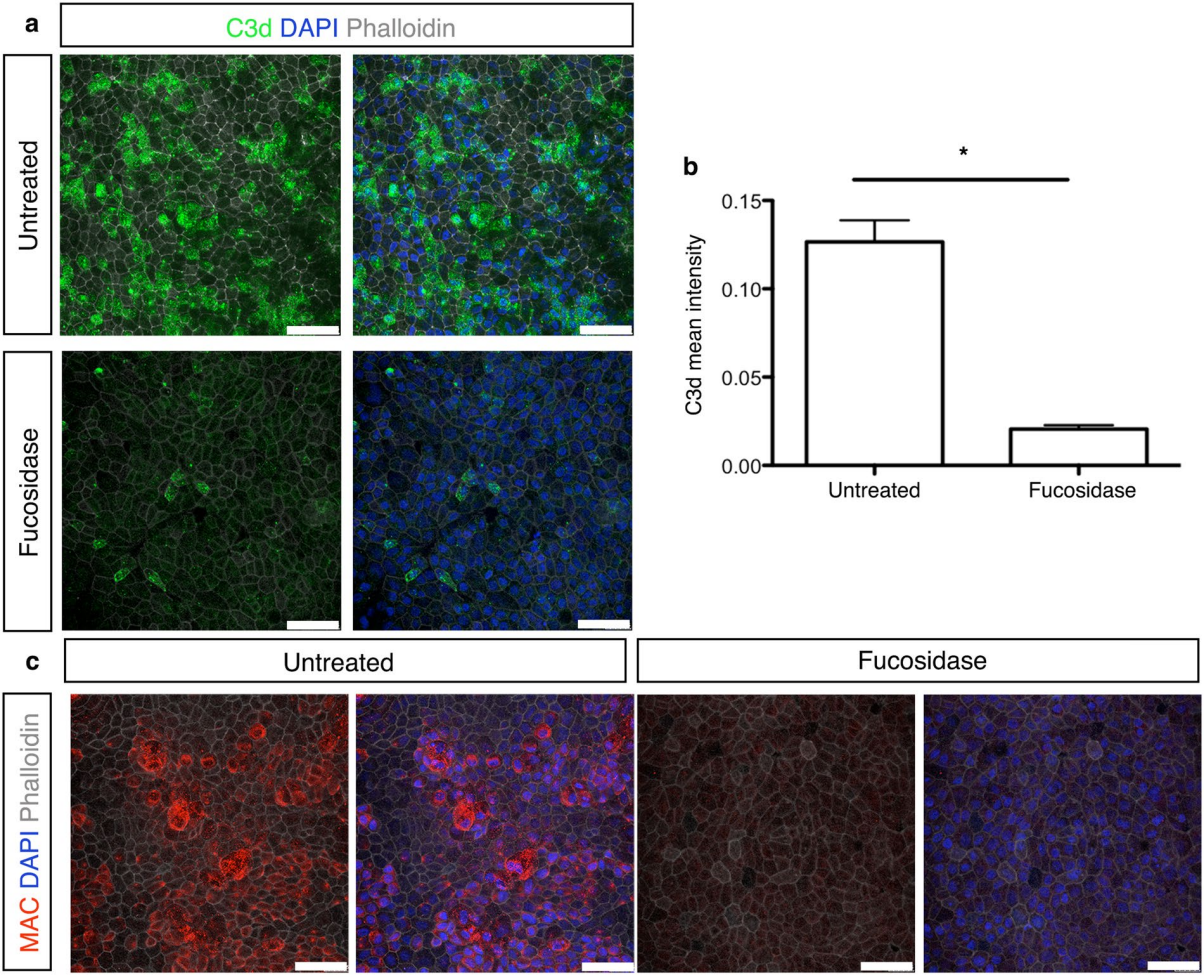
C3d expression and MAC formation on hypoxia-stressed iPS-RPE cells following fucosidase treatment. (**a**) Representative immunofluorescence images showing C3d deposition on the surface of hypoxia-stressed iPS-RPE cells treated or not overnight with fucosidase. C3d (green), nuclei nuclei (blue) and phalloidin (grey) are shown. Scale bars, 50 μm. (**b**) Quantification of C3d expression level on hypoxia-stressed iPS- RPE cells compared to fucosidase-treated hypoxic cells using ImageJ software. (**c**) Representative immunofluorescence images showing MAC staining on the surface of hypoxia-stressed iPS-RPE cells treated or not overnight with fucosidase. MAC (red), nuclei (blue) and phalloidin (grey) are shown. Scale bars, 50 μm.

To better understand the role of L-fucose to mediate CL-11 binding and complement activation, we carried out experiments with hypoxia-stressed iPS-RPE cells treated with fucosidase, as described above (Fig. 3f). As shown in Fig. 5a,b, fucosidase-treatment dramatically decreased C3d deposition and MAC assembly (Fig. 5c) on the surface of hypoxia—stressed iPS-RPE cells. By contrast, untreated cells retained C3d and MAC staining under stress conditions. Taken together with the data represented in Fig. 3g,h, this result supports the notion that CL-11 bound to the surface of hypoxia-stressed iPS-RPE cells acts a trigger for C3d deposition and consequentially causes MAC formation on the surface of hypoxia-stressed iPS-RPE cells.

### CL-11 associated with MASP-2 drives complement activation on hypoxia-stressed iPS-RPE cells

**Figure 6:**
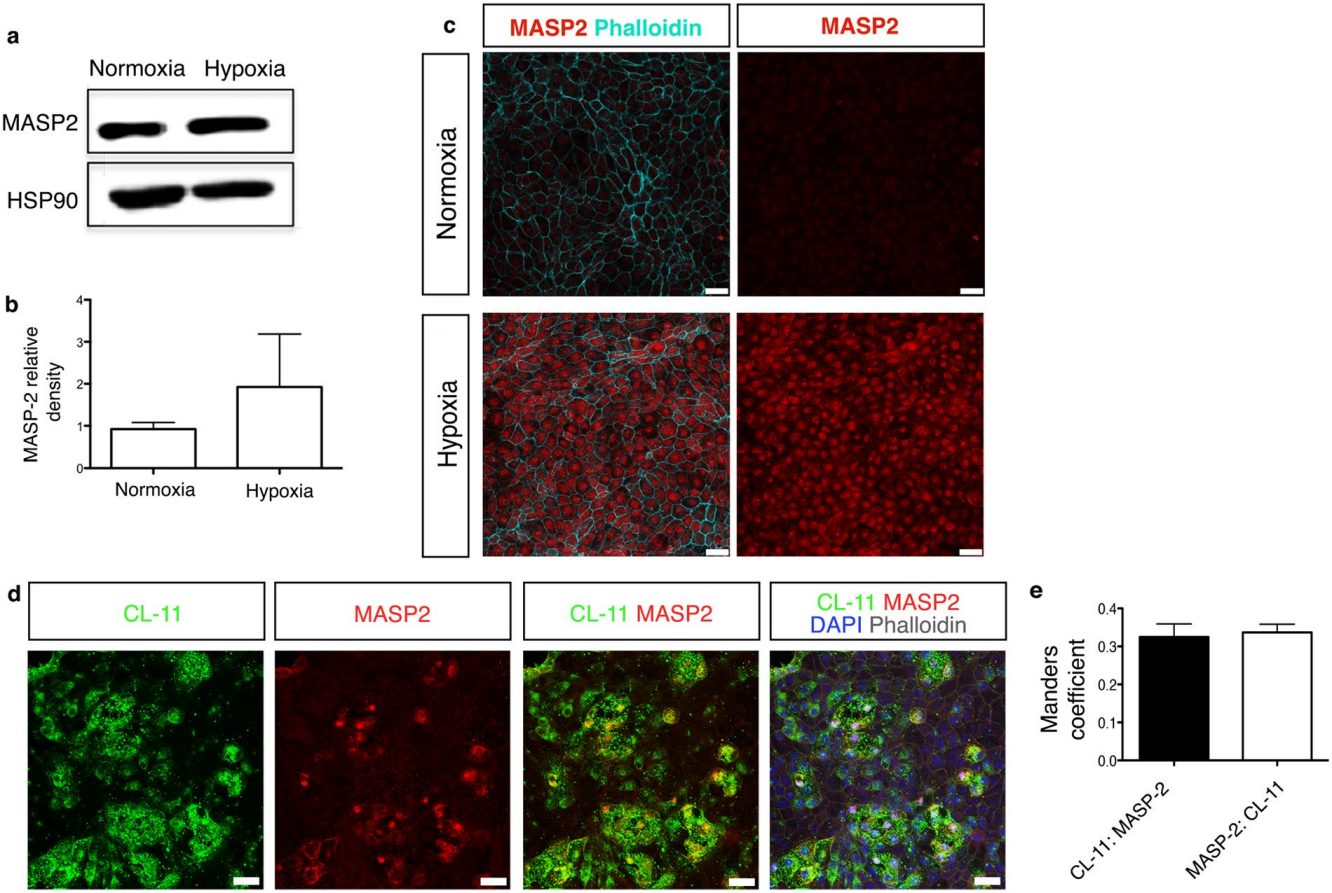
CL-11 and MASP-2 interaction on hypoxia-stressed iPS-RPE cells. (**a**) Representative Western blot showing MASP-2 protein expression in iPS-RPE cells under normal and hypoxic conditions. HSP90 was used as a loading control. Full-length blots are presented in Supplemental Fig. 6. (**b**) Relative density analysis of MASP-2 in iPS-RPE cells under different treatments. N = 3 independent experiments. (**c**) Representative immunofluorescence images showing MASP-2 staining on hypoxia-stressed iPS- RPE cells compared to non-stressed cells. MASP-2 (red) and phalloidin (aqua) are shown on the left panels and MASP-2 (red) alone is shown on the right panels. Scale bars, 25 μm. (**d**) Representative immunofluorescence images showing co-expression of CL-11 and MASP-2 (orange) on the surface of hypoxia-stressed iPS- RPE cells MASP-2 (red), CL-11 (green) nuclei (blue) and phalloidin (grey) are shown. Scale bars, 25 μm. (**e**) CL-11 and MASP-2 co-localization was quantified by calculating Mander’s co-localization coefficients using JACoP plug-in and ImageJ software.

CL-11 circulates as complexes with MBL/ficolin, CL-associated serine proteases, MASP-1, MASP-2 and MASP-3 that, following the binding to pathogens, cleave C4 and C2 to generate the classical C4b2a convertase and initiate complement activation^20,21^. In addition, it has been demonstrated, in models of cardiac, intestinal and renal reperfusion injury, that MASP-2 is a crucial mediator of tissue injury and can cleave C3 independently of C4^47–49^. Under normal culture conditions RPE cells express several complement components and regulators^50^; however the expression of MASP-2 has not previously been reported. We examined whether MASP-2 was expressed in iPS-RPE cells and whether CL-11 used MASP-2 to initiate complement activation on hypoxia-stressed iPS-RPE cells. As shown in Fig. 6a, Western blot analysis disclosed the expression of MASP-2 in iPS-RPE cells; however no major differences were observed in normal cells versus hypoxic cells (Fig. 6b). Interestingly, prominent MASP-2 staining was detected in hypoxic iPS-RPE cells by immunofluorescence compared to non-stressed cells (Fig. 6c). This prompted us to investigate whether CL-11 could interact with MASP-2 on the surface of hypoxia-stressed iPS-RPE cells.

The immunofluorescent images for CL-11 and MASP-2 show there are numerous areas where these two molecules are co-located on RPE cells (Fig. 6d,e), though it is difficult to establish the precise extent of overlap due to the weaker staining of MASP-2 compared to CL-11. Nonetheless, having already shown CL-11 interaction with fucosylated ligand is required to deposit complement (Fig. 4b), the location of MASP-2 at the same place as CL-11 suggests a unifying mechanism in which complement activation on the hypoxia-stressed epithelial cells is dependent on the physical presence of MASP-2 at the CL-11-ligand interaction site.

## Methods

### Ethical statement

Human eye tissue used in this study was provided by Moorfields Biobank with support from NIHR funding. Informed consent was given for all anonymous tissue samples used and ethical approval was given by the NHS Research Ethics Committee (10/H0106/57–14ETR41). All methods were carried out in accordance with guidelines and regulations as licensed by the HTA at the UCL Institute of Ophthalmology.

### iPS-cell derived RPE differentiation culture

Human iPS stem cell lines (IRM90-4 from Wicell) were maintained on feeder free conditions on E8 (Thermo Fisher) and geltrex coated 6 well plates. Briefly, when 80% confluent hiPS were dissociated using a 1:1 dispase and collagenase solution for 10 minutes. iPS clumps were collected, washed twice with PBS and resuspended in E8 media for further maintenance culture on 6 well plates. Human iPS cells were maintained as described above until 90–95% confluent, then media without FGF (E6, Thermo Fisher) was added to the cultures for two days (D1 and 2 of differentiation) followed by a neural induction period (up to 7 weeks) in proneural induction media (PIM; Advanced DMEM/F12, MEM non essential amino acids, N2 Supplement, 100 mM Glutamine and Pen/Strep). Lightly- pigmented islands of RPE appeared as early as week 3 in culture. Media was then changed to retinal differentiation media (RDM; DMEM, F12, Pen/Strep and B27 without retinoic acid) until defined islands of RPE appeared. Pigmented areas of RPE were picked under the dissection microscope using a 21G needle, dissociated by trypsin and plated on laminin (BD Bioscience) coated 24 plate wells in RDM. Once confluent and pigmented cells were expanded up to two times for various experiments. Maintenance cultures of hPSCs were feed daily and differentiation cultures were feed every 2 days.

### Immunohistochemistry

For human retinal sections human eyes collected by Moorfields Lions Eye Bank for corneal transplantation from anonymous donors were fixed in 10% formalin for 48 hours prior to processing and embedding in wax paraffin. 3 μm sections were cut and mounted on slides (Superfrost Ultra plus, Thermo Scientific). Following paraffin removal by Histo-Clear and re-hydration of slides, sections were subjected to antigen retrieval by pressure cooker heating in low pH sodium citrate buffer (Antigen unmasking solution, Vector Labs H-3300). Sections were then blocked for 1 hour in blocking buffer (5% donkey serum) with or without Triton X-100 (2%) in PBS, followed by 1 hour at RT with primary antibodies diluted in blocking buffer. Sections were washed with PBS (2 × 5 minutes) and incubated with secondary antibody diluted in blocking buffer for 1 hour at RT. Sections were then washed in PBS (2 × 5 minutes).

For staining of RPE cells human iPS-RPE cells were cultured either in glass chamber slides or in transwells and placed under hypoxic or normoxic conditions. Cells were fixed for 1 hour in 4% paraformaldehyde, blocked in 5% goat serum and 1% bovine serum albumin in PBS (block solution). Primary antibody was incubated overnight at 4 °C. Sections were incubated with secondary antibody for 2 hours at RT, washed and counter-stained with DAPI (Sigma-Aldrich). Alexa fluor 488 and 546 secondary antibodies (Invitrogen-Molecular Probes) and Alexa fluor 633 Phalloidin (Thermo Fisher Scientific) were used at a 1:500 dilution. The following antibodies were used: mouse monoclonal anti- C3d, (1:100, Abcam); mouse monoclonal anti- C5b-9 (1:100, Abcam); polyclonal rabbit anti-human MASP-2 (1:100, Abcam), mouse monoclonal anti- RPE65 (1:200, Abcam), rabbit polyclonal anti ZO-1 (1:500, Thermo), mouse monoclonal anti-Bestrophin (1:300, Millipore/Merck), mouse monoclonal anti-CRALBP (1:300, Abcam), rabbit polyclonal anti-Otx2 (1:200, Abcam), rabbit polyclonal anti-EZRIN (1:100, Abcam) and rabbit polyclonal anti-HIF2 (1:100, Novus Biologicals).

For hypoxic probe experiments RPE cells were incubated with a 1:1 hydroxic probe (EF5; 10 mM in 0.9% saline solution; University of Pennsylvania, USA) solution in RDM media probe for 24 hours under hypoxia conditions (see below). Control cells were kept under normoxia. After treatment cells were fixed in 4% paraformaldehyde for 1 hour and incubated for 2 hours in blocking solution and incubated overnight in Cy3-conjugated ELK-351 antibody (diluted 1:1 in blocking buffer) at 4 °C. Staining controls (one unstained and one competed stain) were also prepared *as per* manufacturer’s instructions.

For all the above procedures Nuclei were stained DAPI (Sigma-Aldrich) for 5 minutes, washed with PBS and coverslip mounted with fluorescent mounting medium (DAKO, S3023). Images were acquired by confocal microscopy (Leica DM5500Q, Leica Microsystems).

### Induction of hypoxic stress and cell viability

Human iPS-RPE cells were cultured under hypoxic conditions (5% CO_2_, 1% O_2_ and 94% N_2_) in a gas chamber (Stem Cell Technologies) for 24 hours. Cell viability was measured using LIVE/DEAD Fixable Yellow Dead Cell Stain Kit (Thermo Fisher Scientific) according to the manufacturer’s instructions. Cells were analysed by flow cytometry using BD FACS CANTO II (BD Bioscience) and FlowJo software v9.7.5 for Mac.

### Intracellular CL-11 staining in cultured iPS-RPE cells

iPS-RPE cells cultured on chamber slides, were fixed in 4% paraformaldehyde for 1 hour at room temperature and permeabilised with 3% Triton 1% BSA and 5% serum for 2 hours. Cells were blocked in PBS 20% donkey serum (Sigma-Aldrich) for 30 minutes and incubated with a rabbit polyclonal anti-CL-11 antibody (1:100, Abbexa) at 4 °C overnight. The slides were washed three times in PBS and incubated with secondary donkey anti-rabbit Alexa Fluor 488 (1:500, Abcam) or donkey anti-rabbit Alexa Fluor 594 (1:500, Invitrogen-Molecular Probes) for 1 hour at room temperature in the dark, and washed as described above. The nuclei were counterstained with DAPI (Sigma-Aldrich). The cells were washed, coated with mounting media (DAKO, S3023) and analysed by a Leica DM5500Q confocal microscopy.

### Detection of CL-11 binding to cultured iPS-RPE cells

Cultured iPS-RPE cells were subjected to hypoxic stress as described before. Following 24 hours, the cells were washed in PBS and fixed in 4% paraformaldehyde (but not permeabilised) for 1 hour at room temperature. iPS-RPE cells were then stained for CL-11 by immunofluorescence using either rabbit polyclonal anti-CL-11 antibody (1:100, Abbexa) or a mouse monoclonal IgG anti-human CL-11 produced in house. The detection of L-fucose was achieved using Fluorescein *ULEX EUROPAEUS* Agglutinin I (1:40). To remove L-fucose from cell surface, iPS-RPE cells were put under hypoxic conditions for 24 hours, fixed in 4% paraformaldehyde for 1 hour at room temperature and treated then with α1-2,3,4,6 fucosidase (New England BioLabs) at 37 °C overnight.

### Western blotting

iPS-RPE cells were lysed in RIPA buffer (Thermo Fisher Scientific) containing protease inhibitor cocktail (Calbiochem), for 30 minutes on ice and centrifuged for 15 minutes at 14,000 x *g*. Protein concentrations were determined by Quick Start Bradford assay kit (Bio-Rad), according to the manufacturer’s instructions. Protein lysates were denatured by adding 2 × Laemmli buffer (Bio-Rad) containing 5% β-mercaptoethanol (Sigma). Protein samples (15–30 μg) were separated on 10% or 12% sodium dodecyl sulfate-polyacrylamide gels and transferred onto polyvinylidene difluoride (PVDF) membranes (Millipore). Membranes were blocked in 10% non fat dry milk (Bio-Rad) in PBS plus 0.1% Tween-20 for 30 minutes at room temperature and incubated with primary antibodies overnight at 4 °C, followed by three washes with PBS plus 0.1% Tween-20. Detection of the immunoreactive bands was performed with the ECL Western Blotting Substrate (Bio-Rad), and chemiluminescence was detected with the ImageQuant imaging system using anti-rabbit or anti-mouse HRP-linked antibody (Cell Signaling Technology) for 2 hours at RT. Primary antibodies raised against the following proteins were used for Western blotting: CL-11 (Abbexa, rabbit polyclonal), C3 (Abcam, mouse monoclonal), Actin (Santa-Cruz Rabbit polyclonal,), MASP-2 (Abcam, Rabbit polyclonal), HIF-1α (Abcam, Rabbit polyclonal) and HSP90α/β (Santa-Cruz Rabbit polyclonal). Culture supernatants of normal and hypoxic iPS-RPE cells were concentrated 50 times using Vivaspin 6 ml 10,000MWCO spin columns (Sartorius) and analysed for CL-11 expression.

### Flow cytometry

For detection of endogenous CL-11 expression, human iPS-RPE cells and primary murine RPE cells were treated with TrypLE Express enzyme solution (Thermo Fisher Scientific) for 10 minutes and single-cell suspensions were fixed and permeabilised (eBioscience) at 4 °C for 30 minutes. CL-11 staining was performed by preincubation of cells with 20% donkey serum for 30 minutes and then incubated with polyclonal rabbit anti-human CL-11 Ab (1:100), followed by donkey anti-rabbit Alexa Fluor 488 ab. Exogenous CL-11 binding was detected performing CL-11 staining without permeabilising the cells. Stained cells were analysed by flow cytometry using BD FACS CANTO II (BD Bioscience) and FlowJo software v9.7.5 for Mac.

### Statistics

Regarding Immunohistology and Western Blotting, data are shown as mean ± standard error (SEM), unless otherwise noted. A paired, two-tail Student *t*-test was used to compare two groups. *P*-values below 0.05 were considered significant. All analyses were performed using Graph Pad Prism, version 5 (GraphPad Software) and ImageJ software v1.51k for Mac with JACoP plugin.

### Data availability

The datasets generated during and/or analysed during the current study are available from the corresponding author on reasonable request.

## Discussion

While transplantation of pluripotent stem cell derived-RPE cells is a promising approach to treat visual disorders, a number of factors may impede success and these include the immune response against the transplanted cells and in particular the potential impact of hypoxia in the local environment during cell preparation or in the retinal niche. In this study we present evidence that the complement pattern recognition molecule CL-11 is broadly expressed in the human eye and in RPE cells generated from human skin fibroblasts (iPS-RPE cells). It is already known that several complement components and regulators can be expressed locally in the eye and that RPE cells are a major source of local complement expression^51–54^, suggesting that the retina has the capacity both to initiate and control local complement activation. Indeed, gene association studies in patient groups with AMD have indicated that allelic differences in the regulation of complement activation have a significant effect on disease pathogenesis^55–57^. Moreover, several *in vitro* studies have shown that the retinal complement machinery is regulated by inflammatory cytokines and chemokines such as TNF-α, IFN-γ, IL-27^58,59^ or supernatants of macrophages containing proinflammatory factors^50^ indicating that the retinal complement system can actively respond to microenvironmental insults.

Here we demonstrate that cultured iPS-RPE cells have the capacity to produce, release and bind autocrine CL-11 under hypoxic cell stress. Our results also show that CL-11 released by stressed iPS-RPE cells is functionally active, since CL-11 expressed and bound by the cells was able to promote complement activation leading to C3 deposition and MAC formation on the cell surface. These data highlight that, although retinal cells may suppress inflammation under normal physiological conditions, in the presence of tissue insult, retinal cells could contribute to the local inflammatory response. Specifically, the results suggest that hypoxia-induced tethering of CL-11 to iPS-RPE cells can induce an inflammatory phenotype. Previously we have shown that renal epithelial cells of both human and murine origin also exhibit CL-11-dependent activation of complement in response to cell stress, suggesting that inflammatory signalling mediated by CL-11 is a conserved feature of epithelial cells of diverse origin^28^. Further work is needed to determine the potential impact on inflammasome activity of the cells and consequences for cell function during differentiation, antimicrobial defence and – of most relevance here – in the context of possible stem cell therapy applications. Moreover, our data imply that CL-11 driven complement activation may play a role in AMD pathophysiology.

Our data suggest that CL-11 can act in an autocrine or paracrine fashion, inducing iPS-RPE cell injury through complement pore formation and release of the small pro-inflammatory fragments C3a and C5a, presumed to occur following the cleavage of C3 and C5. Although differentiated iPS-RPE cells are morphologically and functionally close to native RPE cells, an important question for future work to resolve involves the extent to which inflammatory phenotype is affected by manipulation of the cells during cell culture or therapeutic administration and whether this will have a significant bearing on cell viability, cell differentiation and cell immunogenicity. Another key question is whether healthy stem cell derived-RPE cells transplanted into an inflamed eye, such as in AMD patients, can be transformed into an inflammatory phenotype mediated by cell-endogenous CL-11, since the chronic retinal ischaemic environment can induce local cell death via complement activation and therefore potentially compromise the outcome of the transplant itself^60–62^. The inflammatory niche of the recipient retina could be important here. For example, adult hepatocytes and islet cells used for transplantation can induce a profound and immediate inflammatory response following contact with human serum, which is thought to involve triggering of the complement and coagulation cascades^63–65^. It will be essential to verify whether stem cell derived-RPE cells have similar properties and whether cell-protective therapy will be appropriate to enhance their utility in this particular indication.

Our current study provides evidence of a fucosylated ligand presented on the surface of the stressed RPE cells that binds CL-11 produced by the cells. Similarly, a fucosylated ligand has been found on adult mouse renal tubule cells also following hypoxic cell stress. This supports the concept of damage-associated carbohydrate recognition pattern(s) detected by CL-11 in different species and tissues. It is known that hypoxic stress induces alterations in the cell surface of endothelial cells, which could activate the complement system through the lectin pathway^66^. Moreover, it has been shown that different rat retinal laminae show specific patterns of expression of glyco-conjugates, suggesting that their functions are common to the specific retinal regions concerned^36^. Resolution of the glycan structure recognised by CL-11 on human tissue is already underway, and it will be interesting to compare the forms on stress-activated cells of different tissue origin, including the retina.

We have previously shown that blockade of the interaction of the L-fucose with CL-11, with either soluble L-fucose or fucosidase, resulted in an extensive reduction of CL-11 binding to stressed cells and complement activation^28^. Here we used the same approach to show that the inhibition of CL-11 binding results in less complement activation on stressed-RPE cells. The finding that the L-fucose is a putative a damage-associated ligand present on the surface of epithelial cells following cell stress, is in line with a recent study showing that fucoidan, a heterogeneous group of sulphated polysaccharide with high content of L-fucose, reduces the secretion of the vascular endothelial growth factor (VEGF)^67^, the most important factor associated with in the development of wet AMD^68^, in retinal cells and the angiogenesis *in vitro*.

Like other collectin molecules, CL-11 uses MASP-2, which is physically associated with CL-11, to cleave C3 and subsequently C5 prior to MAC formation. In fact, MASP-2 activity has been shown to be essential to mediate tissue injury in models of post-ischemic injury of heart, intestine and kidney in a C4-independent manner^47^. Our findings that MASP-2 protein expression occurs in iPS-RPE cells suggests that the iPS-RPE cells used for this study are self-sufficient as a source of complement components that drive complement activation downstream through the formation of the MAC. There is abundant evidence that sublytic amounts of MAC formed on epithelial and other cell surfaces induces inflammasome activation, including the release of inflammatory cytokines, eicosanoids, collagen and other profibrotic factors^69–71^. We have not yet confirmed the extent of this activity and dependence on MAC formation to iPS-RPE cells, but plan to do so. Furthermore it should be noted that complement-independent effector functions also characterise the collectin family of pattern recognition molecules; these include macrophage activation and local immune activation. Therefore it may be expected that CL-11 will have complement-independent actions of relevance to the inflammatory status of RPE cells and potential application in eye disease. In summary, our study suggests that CL-11, a recently described component of innate immunity, is of relevance to the inflammatory status of RPE cells and may thus play an important role in AMD pathogenesis and the outcome of therapeutic interventions.

## Electronic supplementary material

Supplementary data
