# Supplementary material for: Human stem cell-derived retinal epithelial cells activate complement via collectin 11 in response to stress
Source: Sci Rep. 2017 Nov 7;7:14625. doi: 10.1038/s41598-017-15212-z (PMC5677091; doi:10.1038/s41598-017-15212-z)
Supplement: Supplementary file 1 — Supplementary data [file 41598_2017_15212_MOESM1_ESM.pdf]

Supplementary data

Human stem cell-derived retinal epithelial cells activate complement via collectin 11 in response to stress

Giorgia Fanelli[1]; Anai Gonzalez-Cordero[2,4]; Peter J Gardner[2,4]; Qi Peng[1]; Milan Fernando[2]; Magdalena Kloc[2]; Conrad A. Farrar[1]; Arifa Naeem[2]; Peter Garred[3]; Robin R. Ali[2] and Steven H. Sacks[1]*.

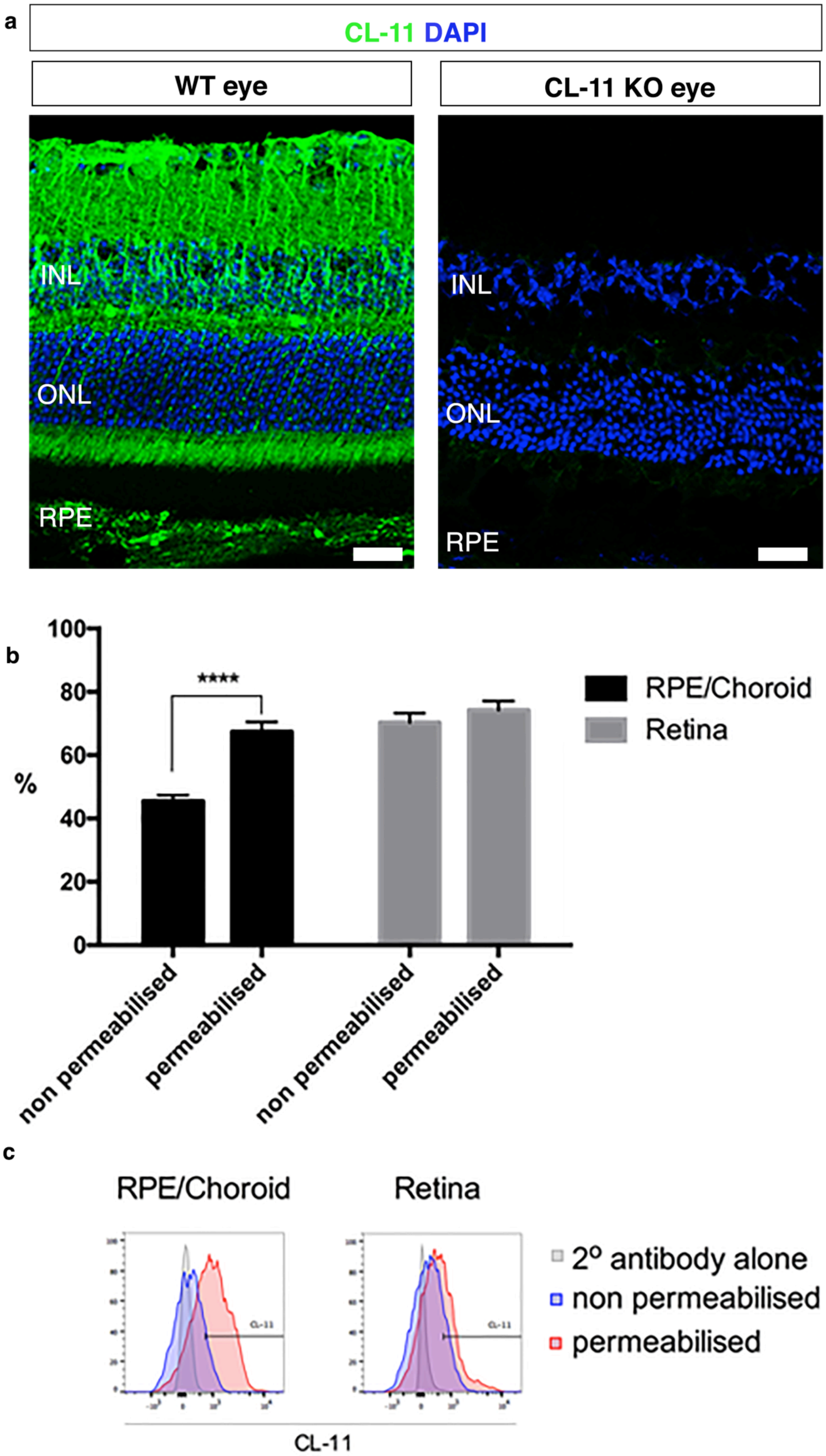
a
CL-11 DAPI
WT eye
CL-11 KO eye
INL
ONL
RPE
INL
ONL
RPE
b
100
80
60
40
20
0
%
****
RPE/Choroid
Retina
non permeabilised
permeabilised
non permeabilised
permeabilised
c
RPE/Choroid
Retina
2º antibody alone
non permeabilised
permeabilised
CL-11

Supplemental Figure 1. Detection of CL-11 expression in mouse retinal sections. (a) Representative confocal image of CL-11 staining (green) in retinal sections from *Colec11*$^{+/+}$ and *Colec11*$^{-/-}$ mouse eye. Scale bars, 25 μm. (b) Quantification of CL-11 expression on permeabilised primary mouse RPE and RPE/Choroid s cells versus non-permeabilised cells. **** P< 0,001, *t*-test. (**c**) Representative flow cytometry histograms showing CL-11 expression on permeabilised (intracellular) versus non-permeabilised (surface) primary mouse Retina and RPE/Choroid cells. Cells stained with secondary antibody alone were used as control.

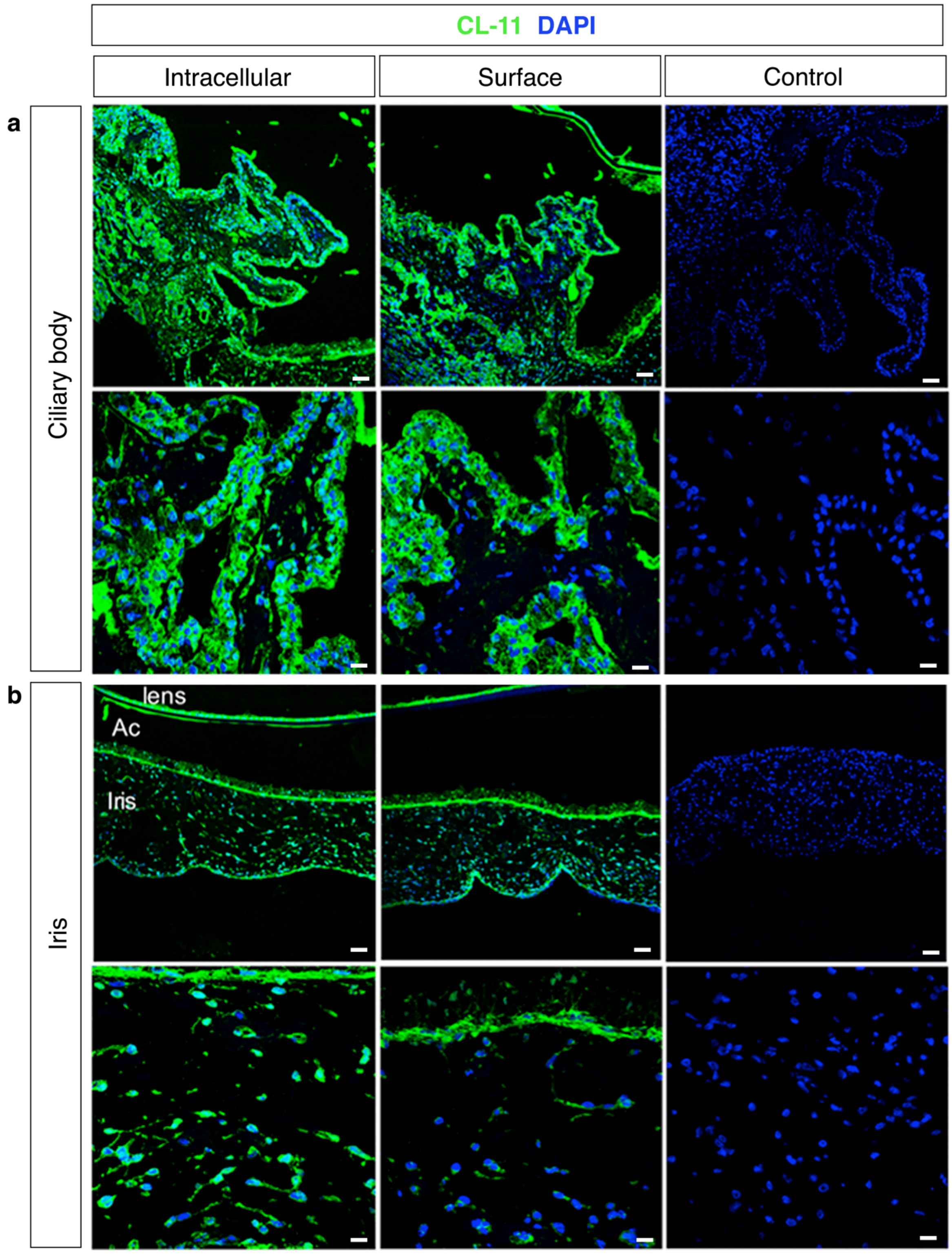

Supplemental Figure 2. CL-11 expression in human eye. Representative confocal image of human eye sections showing intracellular and surface expression of CL-11 in the ciliary body (a) and iris (b). Control sections were incubated with no primary antibody. Scale bars, 25 μm.

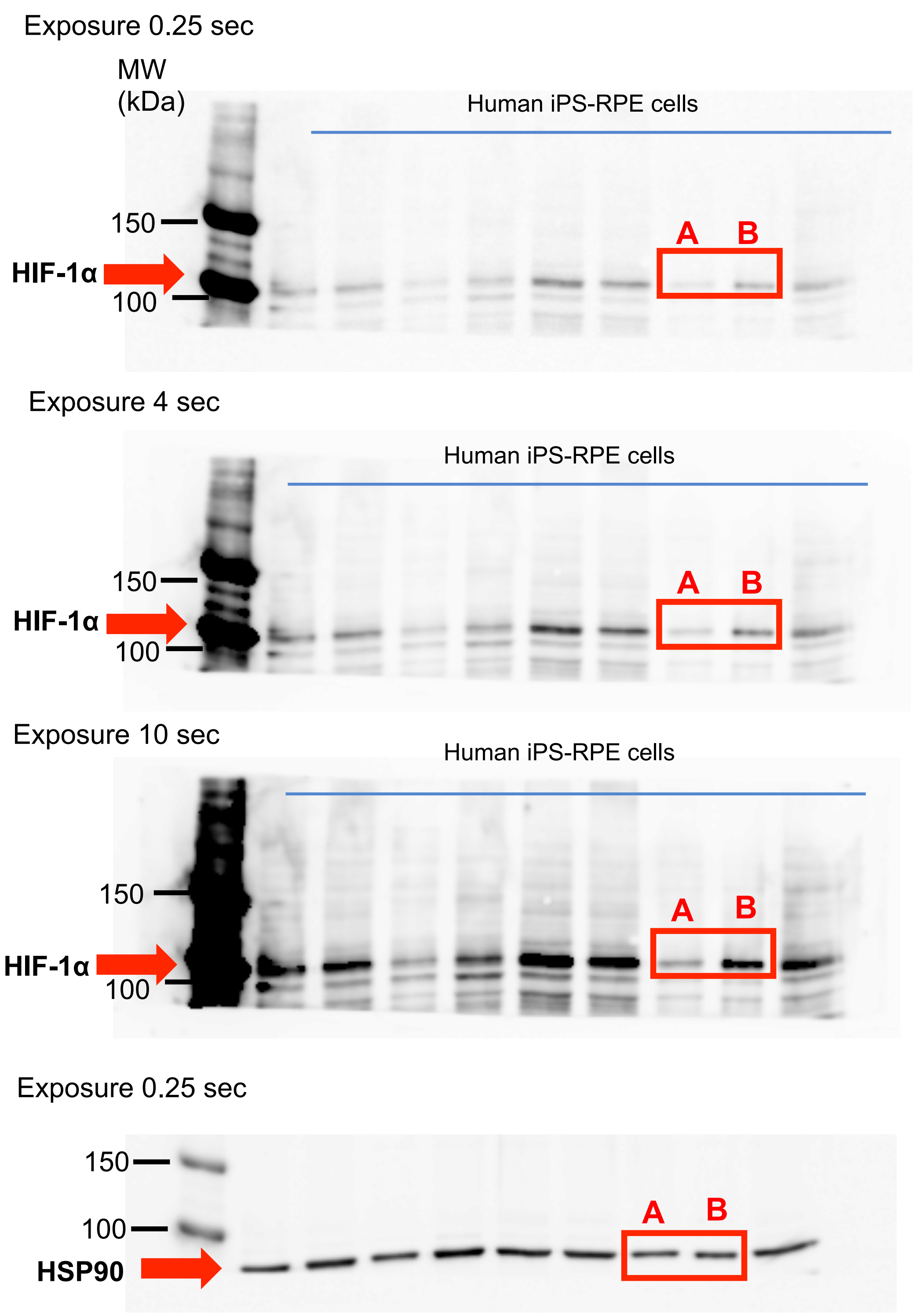

Fig 2c. A, Normoxia; B, Hypoxia

Supplemental Figure 3. Representative full blots of HIF-1α quantification shown in the main figure of the manuscript. The respective figure is indicated

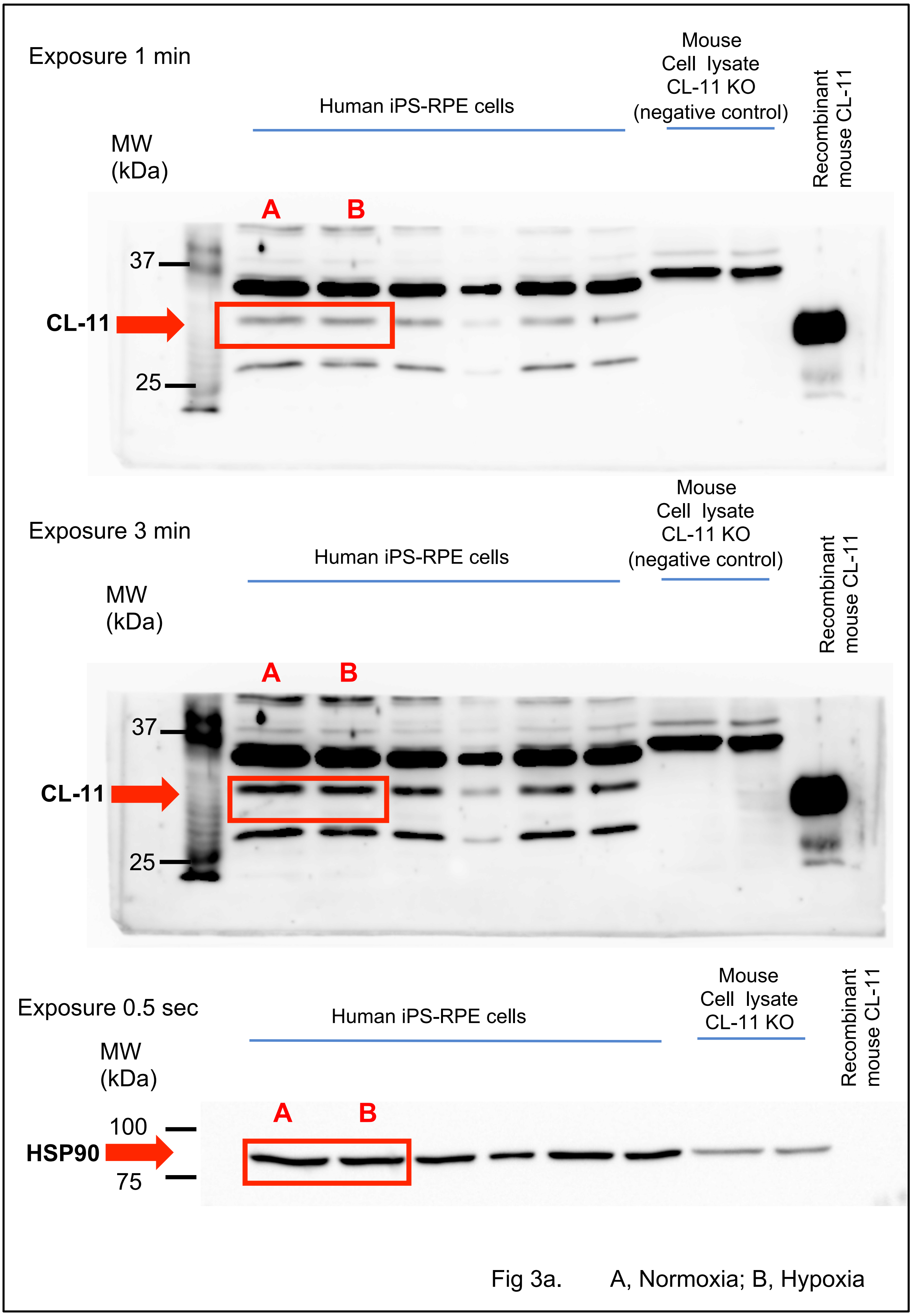

Supplemental Figure 4. Full blots of Immunoblots shown in the main figure of the manuscript. The respective figure is indicated

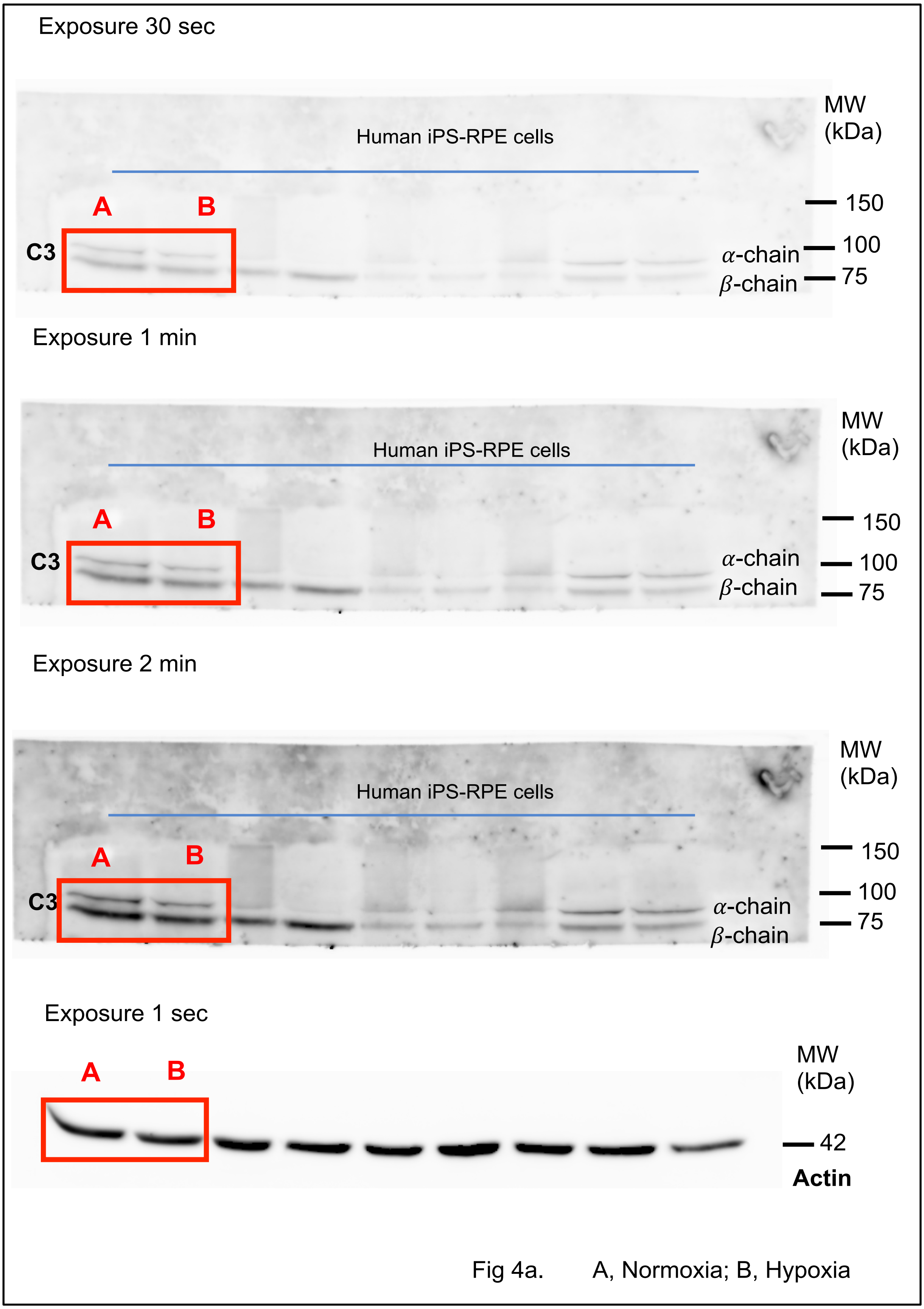

Supplemental Figure 5. Full blots of Immunoblots shown in the main figure of the manuscript. The respective figure is indicated

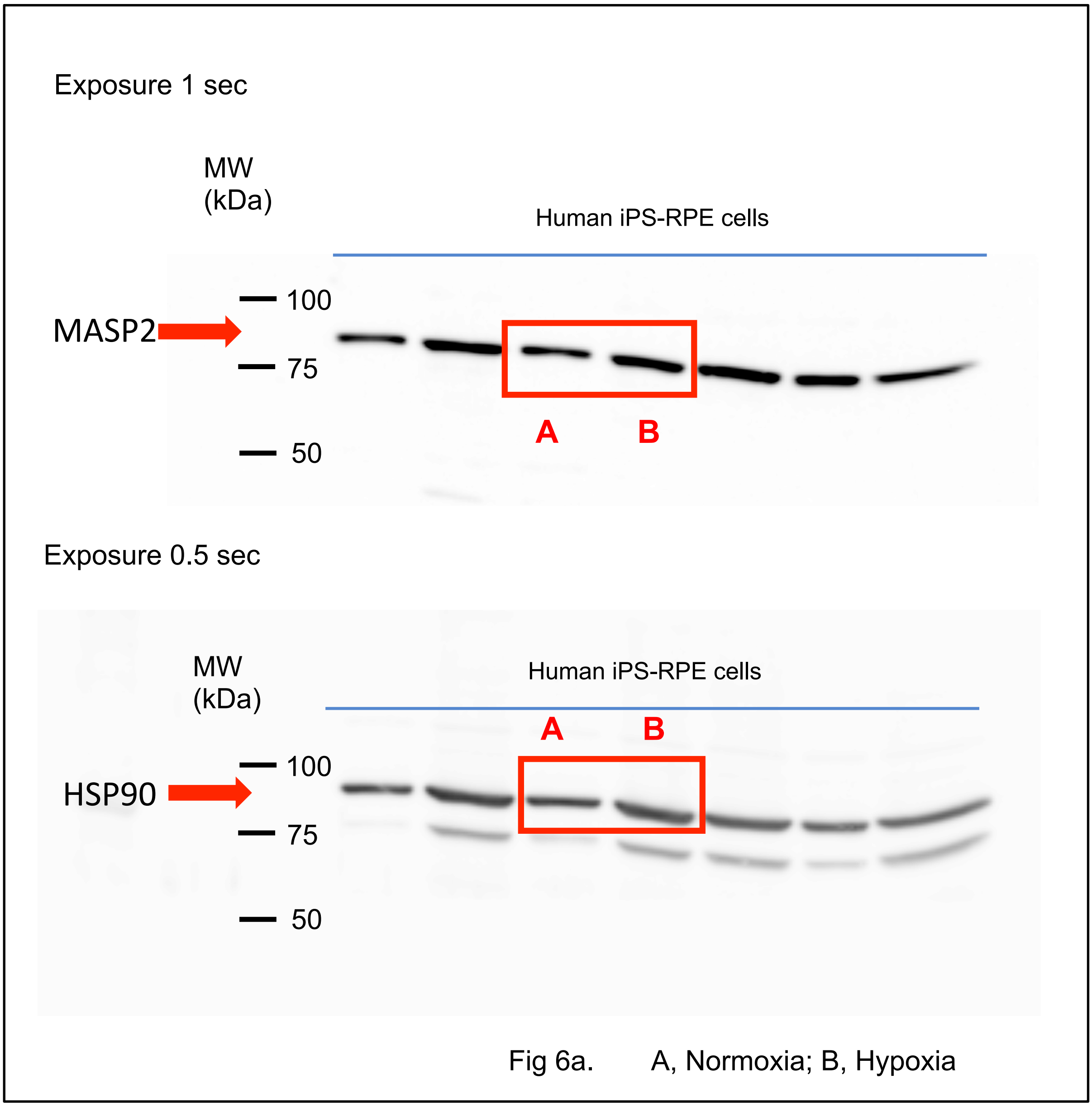

Supplemental Figure 6. Full blots of Immunoblots shown in the main figure of the manuscript. The respective figures is indicated
